# The minimum monitoring signal-to-noise ratio for off-axis signals and its implications for directional hearing aids

**DOI:** 10.1016/j.heares.2017.11.011

**Published:** 2018-01

**Authors:** Alan W. Archer-Boyd, Jack A. Holman, W. Owen Brimijoin

**Affiliations:** a MRC/CSO Institute of Hearing Research (Scottish Section), Glasgow Royal Infirmary, 10-16 Alexandra Parade, Glasgow, G31 2ER, UK; b MRC Cognition & Brain Sciences Unit, 15 Chaucer Road, Cambridge, CB2 7EF, UK

## Abstract

The signal-to-noise ratio (SNR) benefit of hearing aid directional microphones is dependent on the angle of the listener relative to the target, something that can change drastically and dynamically in a typical group conversation. When a new target signal is significantly off-axis, directional microphones lead to slower target orientation, more complex movements, and more reversals. This raises the question of whether there is an optimal design for directional microphones. In principle an ideal microphone would provide the user with sufficient directionality to help with speech understanding, but not attenuate off-axis signals so strongly that orienting to new signals was difficult or impossible. We investigated the latter part of this question. In order to measure the minimal monitoring SNR for reliable orientation to off-axis signals, we measured head-orienting behaviour towards targets of varying SNRs and locations for listeners with mild to moderate bilateral symmetrical hearing loss. Listeners were required to turn and face a female talker in background noise and movements were tracked using a head-mounted crown and infrared system that recorded yaw in a ring of loudspeakers. The target appeared randomly at ± 45, 90 or 135° from the start point. The results showed that as the target SNR decreased from 0 dB to −18 dB, first movement duration and initial misorientation count increased, then fixation error, and finally reversals increased. Increasing the target angle increased movement duration at all SNRs, decreased reversals (above −12 dB target SNR), and had little to no effect on initial misorientations. These results suggest that listeners experience some difficulty orienting towards sources as the target SNR drops below −6 dB, and that if one intends to make a directional microphone that is usable in a moving conversation, then off-axis attenuation should be no more than 12 dB.

## Highlights

• Investigated the minimum signal-to-noise ratio (SNR) required to localize a target.
• Head movement to targets at varying SNRs and locations was measured.
• Orienting towards a new off-axis target became difficult below −6 dB SNR.
• An ideal directional microphone should not attenuate off-axis sources by > 12 dB.

## 1 Introduction

Recent advances in inertial sensor technology and hearing-aid signal processing have made it possible for hearing aids to adaptively change microphone directionality in response to a user's head movements (Boyd et al., 2013, Archer-Boyd et al., 2015, Zohourian et al., 2015). In order to make such technology useful it would be of great benefit to understand what makes an optimal directional microphone that maximizes the signal-to-noise ratio (SNR) for current target sources, while still allowing listeners to detect and orient towards new targets of interest. However, very little is known about the SNR required by a listener to successfully detect and orient towards an off-axis sound source. We call this new metric the Minimum Monitoring SNR. Our investigations provide known constraints for an optimally directional microphone, maximizing the SNR for an on-axis target, while still allowing listeners to accurately and rapidly turn towards a new, off-axis target.

Under ideal conditions, non-adaptive directional hearing-aid microphones can improve the SNR, leading to an improvement in listeners' speech intelligibility in noise scores compared to their scores with omnidirectional microphones (Valente et al., 1995, Bentler, 2005). This benefit is known as the *directional benefit* (Hornsby and Ricketts, 2007). Ideal conditions include a target source that is directly in front of the listener and a noise source in the rear hemifield, and reverberation times of no more than 0.7 s (Ricketts and Dhar, 1999). Directional benefit can be reduced by an increase in reverberation time (Hawkins and Yacullo, 1984) and an increase in source-listener distance in a reverberant room (Ricketts and Hornsby, 2003). If the angle between the target source and the listener's acoustic midline is greater than 60°, the directional benefit becomes a *deficit*, with large reductions in both word recognition scores and pure tone detection beyond 90° (Kuk et al., 2005). An analogous pattern is observed for directionally aided listeners when orienting to targets more than 90° off axis, who make longer and more complicated movements than they do when using omnidirectional microphones (Brimijoin et al., 2014). An optimally directional microphone could reduce the directional deficit for off-axis targets, while preserving as much as possible the directional benefit for on-axis targets.

Non-adaptive directional microphones can significantly improve front-back localization over omnidirectional microphones, as the level difference introduced by the directional microphone response pattern provides an additional cue to the listener (Keidser et al., 2006). However, directional microphones do not improve localization accuracy in general over omnidirectional microphones. Keidser et al. (2006) showed that the mean left/right error was not significantly different between directional and omnidirectional settings. Adaptive directional microphones, where the directional response pattern changes in order to best suppress background noise, have been found to reduce localization performance relative to omnidirectional microphones when tested with a speech-weighted noise source at ±90° and 0 dB SNR (Van den Bogaert et al., 2006). However, at high SNRs of +10 dB, adaptive systems can allow listeners to localize at least as well as when using omnidirectional microphones, and their localization of sounds in the rear hemifield is improved in quiet and noise (Chung et al., 2008). One possible reason for the reduction in performance associated with adaptive systems is their reduced ability to follow a noise source as it becomes more diffuse (Bentler et al., 2004), meaning that the directional pattern of the microphone may change unpredictably at each ear, altering the inter-aural level difference (ILD) cue.

The trade-off between the benefits and deficits associated with directional hearing aids can be revealed in user surveys. In everyday life, hearing-aid users may encounter different acoustic environments and social situations for which a directional microphone may be more beneficial than an omnidirectional microphone, and vice versa (Cord et al., 2004). The factors that have been considered to influence this choice are similar to those considered in the laboratory, including reverberation, and the location of the target source and noise source(s).

The performance of a hypothetical optimally directional hearing aid may be limited by the localization abilities of the user. In general, hearing-impaired (HI) listeners are worse than normal-hearing (NH) listeners when localizing single sources in quiet, although this decrease in performance is only moderately predictable from sensorineural hearing loss, suggesting that audibility is only one factor influencing performance (Noble et al., 1994). If audibility is restored, there is very little difference in horizontal localization ability between NH and HI listeners with losses of up to 50 dB HL (Byrne et al., 1992). Listening in noise is different, however; noise induces a greater detrimental effect on the ability to localize click trains for HI listeners than for NH listeners (Lorenzi et al., 1999), with the effect largest at ±90°. When localizing a target speaker in a spatially separated multi-talker mixture, HI listeners are again significantly worse than NH listeners at localizing an individual talker, despite performing similarly in quiet (Best et al., 2011). In both cases, audibility accounted for some but not all of the reduction in performance, suggesting that other factors related to sensorineural hearing loss are involved. Reduced spectrotemporal sensitivity could be a factor, leading to simultaneous speech sounds masking each other more and a reduced ability to direct spatial attention to a target.

It has been found that sound source localization accuracy can be improved by utilizing head movements (Noble, 1981, Perrett and Noble, 1997a, Perrett and Noble, 1997b). One hypothesis is that these head movements occur in order to place the sound in front of the listener, reducing the minimum audible angle and therefore increasing location discrimination (Blauert, 1997, Begault, 1994). More recent work using 3D virtual audio has found that azimuthal head movements of more than 32° improve elevation localization but have no effect on azimuthal accuracy (McAnally and Martin, 2014). Listeners move their heads constantly, particularly when communicating (Morency et al., 2005), and listener head movements have been investigated in the context of resolving front-back confusions (Wightman and Kistler, 1999, Brimijoin and Akeroyd, 2012, Kim et al., 2013) and auditory externalization (Begault et al., 2001, Brimijoin et al., 2013, Hendrikx et al., 2017). However until recently, little attention has been paid to the types of head movements that are made by HI listeners in order to orient towards a sound. Brimijoin et al. (2010) found that HI listeners exhibit more complex movements in an auditory orientation task than NH listeners, and are generally slower to start to orient towards and finally fixate on a source. A later study investigated the effect of directional and omnidirectional microphones on performance in an auditory orienting task (Brimijoin et al., 2014). For large off-axis target angles, listener movements were more complex when using directional microphones, with listeners taking longer to reach their targets, frequently turning in the wrong direction initially, and rapidly changing their rotational velocities. For smaller angles, however, the pattern was reversed and listeners made simpler and faster movements towards an off-axis target when using a directional setting. This suggests that there is a benefit in using a directional microphone for small angles of movement, and any possible improvements provided by an optimally directional microphone would be measured when orienting towards off-axis sources at large angles.

In the current study, we changed the presentation angle, overall background noise level, and SNR of a speech source in order to find the minimum SNR required for listeners to reliably orient towards that source. Since listeners were not required to understand the speech, but simply orient towards it, we have called this metric the minimum *monitoring* SNR. We recorded head motion and derived a number of measures, such as fixation error and movement duration, in order to gain a better understanding of listeners' behaviour when orienting towards a source and the effect of changing presentation angle, background noise level and SNR on it.

The study was designed to be similar to a real dynamic group conversation in a noisy environment. In these environments, the background noise can vary, and the SNR of individual sound sources may vary due to changing source or background level. Listeners may be required to orient towards current or new group members as the conversation shifts to a new talker. This means that source angles relative to the listener could be in both the front and rear hemifields.

The results of this study are used to make suggestions for future directional hearing-aid design. The minimum monitoring SNR provides a baseline for the SNR at which listeners will still be able to reliably orient towards an off-axis source. Head (and eye) guided directional microphone systems are currently in development. Our analysis of a number of head-motion metrics provides information that could be utilized by these systems to improve their performance with respect to amplifying the sound source of interest and sufficiently suppressing others.

## 2 Materials and methods

### 2.1 Apparatus

The experiment was undertaken using a circular 24-loudspeaker (Tannoy VX-6) ring with a 1.75 m radius in a sound-dampened chamber. Sound presentation was controlled using Matlab and a MOTU 24 I/O soundcard. The loudspeaker array was calibrated daily as follows: a 114 dB calibrator (Norsonic Nor1251) was placed on our reference microphone (G.R.A.S. 46AE) in the center of the loudspeaker ring. The output of the calibrator was captured on a MADI audio interface (RME HDSPe MADI FX and Ferrofish A-16) and its level in volts at 1 kHz was measured, yielding a volt-per-dB value for the microphone. Swept sine signals (0.1–20 kHz) of known output voltage were then presented from each loudspeaker in turn and these signals were captured by the same reference microphone. A comparison between the output RMS level in volts and the converted input level in dB allowed us to compute a dB-per-volt value for each loudspeaker. Finally, the signals for the experiment were played out and their actual average output levels were measured with a B&K sound level meter (B&K 2260) and a fine-tuning offset value was saved alongside the dB-per-volt value and used for all subsequent audio presentations, ensuring the ability to present calibrated signals in dB SPL from each loudspeaker. The Tannoy VX-6s exhibited little variation in frequency response from loudspeaker to loudspeaker. Head movements were recorded using a head-mounted crown and infrared system. The system utilized a Nintendo Wii games controller suspended above the listener to measure yaw. The “crown” worn by the listener had an LED light bar attached to measure via infrared where the listener was facing, at a sample rate of 100 Hz (Brimijoin et al., 2013).

### 2.2 Stimuli and procedure

Listeners sat in the center of the ring and were presented with 24 channels of constant, uncorrelated speech-shaped background noise. Each trial consisted of a reference, presented directly in front of the listener at the beginning of each trial, and a target, presented at a randomly selected angle and level relative to the reference. Listeners were asked to listen for the target amongst the background noise and orient towards it by turning their heads and bodies in a rotating chair, to face exactly where they felt the target source was, and then to press a handheld remote control. The reference consisted of speech from a single male talker and the target stimuli consisted of speech from a female talker, both drawn from the adaptive sentence list corpus (MacLeod and Summerfield, 1990). All signals were presented from angles defined with respect to the instantaneous head angle of the listener at the start of the trial. This was achieved using the motion tracking system described above to capture the head angle and the use of sine/cosine panning for presenting signals at locations between a pair of loudspeakers. The reference was always presented directly ahead of the listener (defined here as 0°) at the beginning of each trial. The target was presented at an angle relative to the position of the reference signal. Targets were presented randomly at either ±45, 90 or 135° from the trial start point. The level of the reference was 70 dB SPL. In one condition, the level of the background noise was −6 dB below the level of the reference. Target levels were −24, −18, −12, and −6 dB relative to the reference level. In another condition the background level was −12 dB relative to the reference level, to examine absolute level effects, as the presentation level of the target was also defined relative to that of the reference level. All conditions were randomized in order.

Each trial began with the reference presented for a duration drawn from a uniform distribution between 1 and 3 s. The reference then stopped and the target was turned on for 6 s. The reference offset was used to cue the listener to begin to search for the target, a particularly important feature since many tested SNRs could make the onset of the target difficult to hear. The experiment started with a practice block consisting of 12 trials. The full experiment consisted of 192 trials split up into 4 blocks with a short break in between each. This was done to mitigate the effects of listener fatigue. Each block was resumed by the experimenter once the listener was ready, with care being taken not to alter the position of the head mounted crown during breaks. On average each trial lasted less than 8 s because the listener ended a trial by pressing the button.

There are two main options for discussing and displaying the data: 1) plot as a function of absolute level, or 2) plot as a function of SNR. We opted for the latter, as the main objective was to investigate the effect of changing the SNR, and plotting the results and conducting the analysis with respect to SNR aided our interpretation of the results. This means that the target SNRs ranged from −18 to 0 dB when the background noise was presented at −6 dB, and from −12 to +6 dB when the background noise was presented at −12 dB. In the resulting plots (Fig. 2, Fig. 3, Fig. 4, Fig. 5, Fig. 6) the absolute level of the target at a given target SNR in −12 dB background noise is 6 dB *lower* than for a target at the same SNR in −6 dB background noise.

### 2.3 Listeners

35 (12 female) HI listeners were recruited. One listener was excluded due to a large asymmetry in their hearing loss, and another due to inaudibility of the stimuli. This left 33 (12 female) HI listeners who were included in the results and analysis. The mean better-ear four-frequency (0.5, 1, 2, 4 kHz) average was 28 dB HL with a range of 7.5–61.3 dB HL. Average asymmetry was 6.6 dB HL. Their average age was 65 (±11 standard deviation) years, ranging from 39 to 81 years.

9 listeners were bilaterally aided, 10 were unilaterally aided and the remaining 16 wore no hearing aids. Mean duration of hearing-aid use for those with hearing aids was 8.2 years with a range of 2–20 years.

**Fig. 1:**
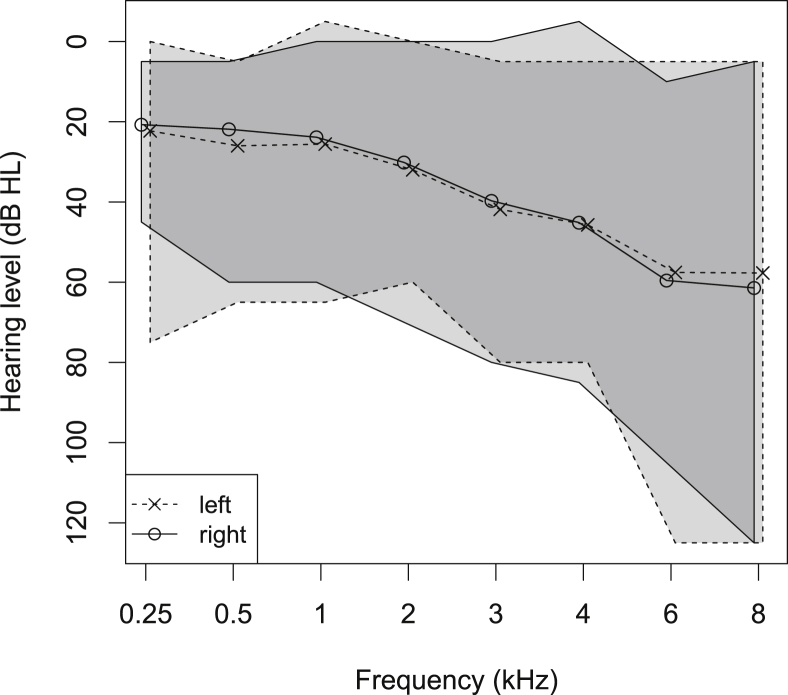
Hearing-impaired participant average audiogram and range. ‘x’ and ‘o’ indicate the left and right ears, respectively. The shaded area bounded by the dashed and solid lines indicate the range of audiograms for the left and right ears, respectively.

Fig. 1 shows the average left and right audiograms of the listeners. The grey areas show the range over which the individual audiograms varied. There was a large variation in listener audiograms and the losses were approximately symmetric.

Some of the lower target level conditions were too quiet for some listeners to hear. To remove these data points from the analysis, we set the criterion that the sensation level for a given target level be at least 5 dB. This was calculated by converting the presentation level in dB SPL to dB HL by subtracting the average difference between dB SPL and dB HL across the frequencies 500, 1000, 2000, and 4000 Hz (10.5 dB), and ensuring that the listener's average better-ear average sensation level (measured across the same frequencies) was at least 5 dB. The number of listeners that met the criterion for each condition are given in Table 1. We discuss the implications of this in the discussion section.

**Table 1:** The number of listeners (N) included for each target SNR, background and target level.

Target SNR (dB)	Background level	Target level (dB)	N
6	−12	−6	33
0	−6	−6	33
0	−12	−12	28
−6	−6	−12	28
−6	−12	−18	22
−12	−6	−18	22
−12	−12	−24	19
−18	−6	−24	19

### 2.4 Deriving metrics from listener motion

All metrics were based on a listener's yaw angle, i.e., rotation about the Z, or vertical, axis. The metrics were: fixation error, movement duration, reversal count, initial misorientation count, and front-back confusion count. Fixation error was computed as the absolute angular difference (in degrees) between the target direction and the listener's head orientation at the end of a trial. Movement duration was computed as the time between the onset of the target and when the listener pressed the ‘finished’ button. Reversal count was defined as the number of instances when a listener's head movement reversed in direction by at least 3°. Initial misorientations were defined as a trial during which the listener initially turned more than 3° in the wrong direction. Finally, front-back confusions were defined as cases where the listener ended their orienting movement more than 150° away from the target direction.

### 2.5 Statistical analysis

The majority of the metrics produced data that were significant in Shapiro-Wilk tests, meaning that the data were not normally distributed. Levene's test was significant, meaning that sphericity assumptions were also violated. Donaldson (1968, in Field et al., 2012) has shown that the F-statistic (or χ^2^ in the case of the linear mixed-effect models used) is robust to violations of normality. Based on this we used linear mixed-effect (LME) models. In order to increase the robustness of any post-hoc tests, trimmed means (20%) and bootstrapping (number of bootstrap samples, n = 2000) were used (Wilcox, 2005), in conjunction with Bonferroni corrections to minimize the Type I error.

In order to compare the effect of level at the same SNR across the recorded metrics, only target SNRs from −12 to 0 dB were included in the LME models, as these target SNRs were used for both the −12 dB and −6 dB background noise levels.

Linear mixed effect (LME) models were built in the same order for each measured variable, starting with a baseline model containing no predictors that assumed the data are part of a random distribution. The variables target angle, target SNR and background level were then added to the LME to investigate main effects, followed by 2-way interaction effects of background level and target angle, background level and target level, and target angle and target level. The final model included a 3-way interaction effect of target angle, target level, and background level. A 1-way ANOVA was used to compare each model to the previous version, given in the form χ^2^(Δdf), p, where χ^2^(Δdf) is the chi-squared score between two models, with increase in degrees of freedom Δdf, and *p* is the p-value. Contrasts are reported with the form *b*, *t*(df), *p*, *r*, where *b* is the contrast value, *t(df)* is the t-value with *df* degrees of freedom, *p* is the p-value, and *r* is the effect size. Contrasts were calculated for angle (45° vs 90°; 135° vs 90°), SNR (−12 dB vs −6 dB; 0 dB vs −6 dB), and background (−12 dB vs −6 dB), and interactions between these variables. Bonferroni-corrected post-hoc trimmed-mean, bootstrapped t-tests are reported as Y_t_(−CI, +CI), *p*, where Y_t_ is the robust t-value, quoted with lower and upper confidence interval (−CI, +CI) values, and *p* is the p-value. If a confidence interval crossed zero, the *t*-test was not significant.

## 3 Results

### 3.1 Fixation error

**Fig. 2:**
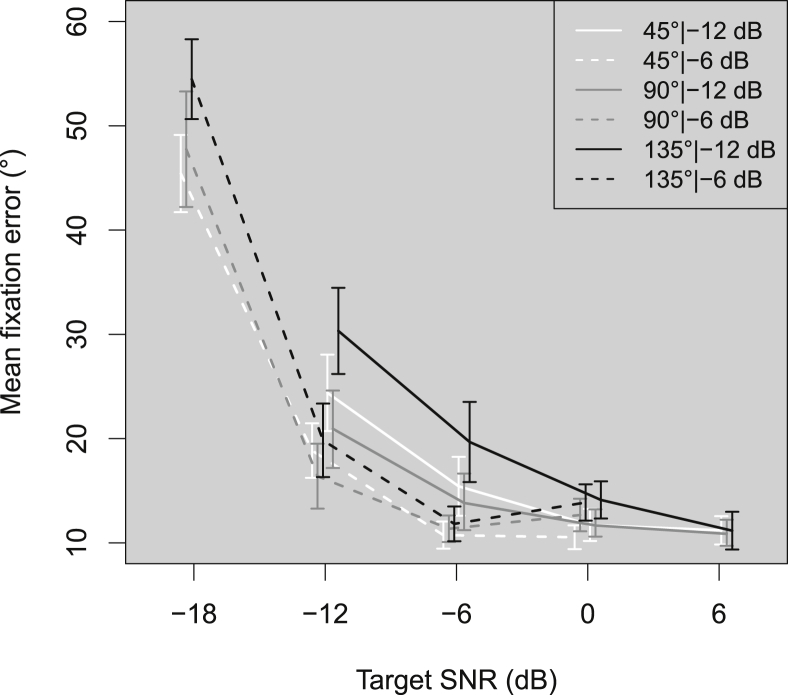
Mean fixation error across participants as a function of the target SNR. Fixation error is the absolute angular difference (in degrees) between the target direction and the listener's head orientation at the end of a trial. White, grey, and blacklines show results for the target at 45°, 90°, and 135°, respectively. Solid lines and dashed lines show results for the background noise at −12 dB and −6 dB relative to the 0° reference. Error bars show ±1 standard error of the mean.

Fig. 2 shows the average fixation error (in degrees) across listeners as a function of target SNR for each combination of target angle and background level. Overall, the fixation error increased with decreasing target SNR. At positive SNRs, the fixation error did not change with target angle or background level. Between 0 and −6 dB SNR, performance did not differ for target angles 45° and 90° at both background levels. At −6 dB target SNR and 135° target angle, a decrease in level increased the fixation error. Between −6 and −12 dB target SNR, the fixation error increased across all conditions and below −12 dB target SNR performance decreased more rapidly. The increase in fixation error from −12 to −18 dB SNR was significant across all target angles (Bonferroni-corrected *p* = 0.05/3 = 0.0167): 45° (Y_t_ = 30.11 (18.16, 42.06), *p* < 0.001); 90° (Y_t_ = 35.41 (13.47, 57.35), *p* = 0.0015); 135° (Y_t_ = 38.89 (28.89, 48.89), *p* < 0.001). The results showed that listener performance started to be negatively affected when the target SNR was between −12 and −6 dB.

1-way ANOVAs of LME models fitted to the fixation error data revealed main effects of target angle (χ^2^(2) = 7.51, *p* = 0.023), target SNR (χ^2^(2) = 53.01, *p* < 0.001), and background level (χ^2^(1) = 10.84, *p* < 0.001). Critically, there was also a significant interaction between background level and target SNR (χ^2^(2) = 13.43, *p* < 0.0012), meaning that there was some additional effect on fixation error when both target SNR and level decreased, in comparison to changing only one of these factors.

Contrasts revealed that the main effect of angle was driven by the difference between 90° and 135° (*b* = 2.30, *t*(64) = 3.23, *p* = 0.002, *r* = 0.37) only. This suggests that fixation accuracy was similar for angles in the front hemifield and only became worse when sound sources were presented in the rear hemifield of the listener.

### 3.2 Movement duration

**Fig. 3:**
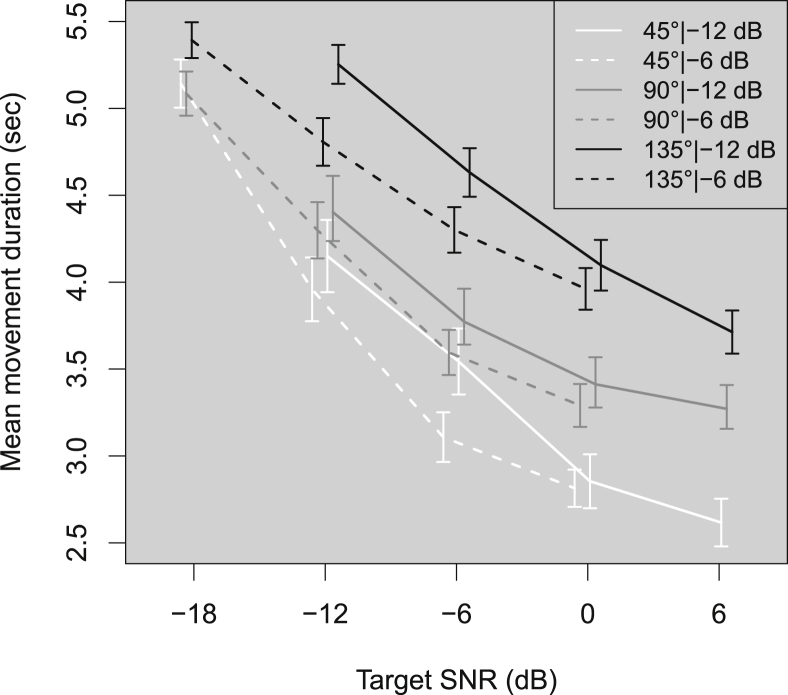
As Fig. 2 but for mean movement duration (seconds). Movement duration is the time between the onset of the target and when the listener pressed the ‘finished’ button.

Fig. 3 shows the mean movement duration in seconds across listeners as a function of target SNR for each combination of target angle and background level. As might be expected, at high SNRs there was an effect of target angle, the listeners taking longer to turn to targets that were further away. At negative SNRs, lower background levels were associated with increased movement durations. At the lowest SNR, the mean movement duration across all target presentation angles was similar, at just over 5 s. The largest increase in movement duration as the SNR decreased was found for targets presented at 45°, the smallest angular distance measured.

1-way ANOVAs of LME models fitted to the movement duration data revealed main effects of target angle (χ^2^(2) = 105.01, *p* < 0.001), target SNR (χ^2^(2) = 182.71, *p* < 0.001), and background level (χ^2^(1) = 18.60, *p* < 0.001). No interaction effects were observed. As the target was presented further away in angle, or at levels or SNRs that made it more difficult to hear, movement duration increased at an approximately constant rate.

### 3.3 Reversals

**Fig. 4:**
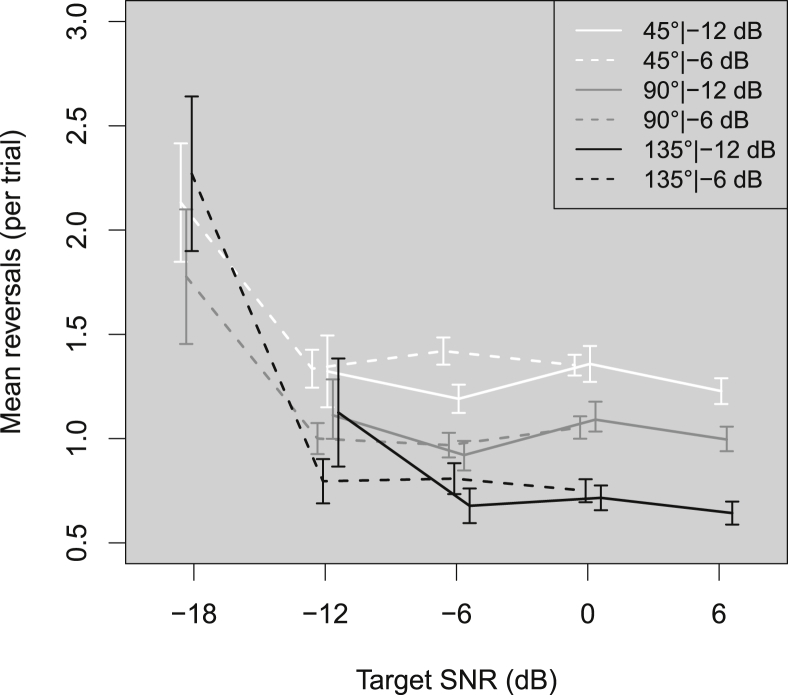
As Fig. 2 but for mean reversals (per trial). Reversals are defined as the number of instances when a listener's head movement reversed in direction by at least 3°.

Fig. 4 shows the mean reversals (per trial) across listeners as a function of target SNR for each combination of target angle and background level. Above −12 dB SNR, reversals were approximately invariant with SNR and were most common when the target angle was 45° and least common when it was 135°. At −18 dB SNR the number of reversals per trial doubled for the 135° target angle, while the increase at 45° and 90° was smaller. Visual inspection showed that the largest change in reversals due to decreasing SNR occurred when the SNR dropped below −12 dB.

1-way ANOVAs of LME models fitted to the fixation error data revealed a main effect of angle (χ^2^(2) = 75.83, *p* < 0.001). Critically, there was a significant interaction effect between background level and SNR (χ^2^(2) = 7.73, *p* = 0.021). Contrasts revealed that the interaction was driven by the change in SNR from −12 to −6 dB ((*b* = 0.073, *t*(198) = 2.67, *p* = 0.0082, *r* = 0.19).

### 3.4 Initial misorientations

**Fig. 5:**
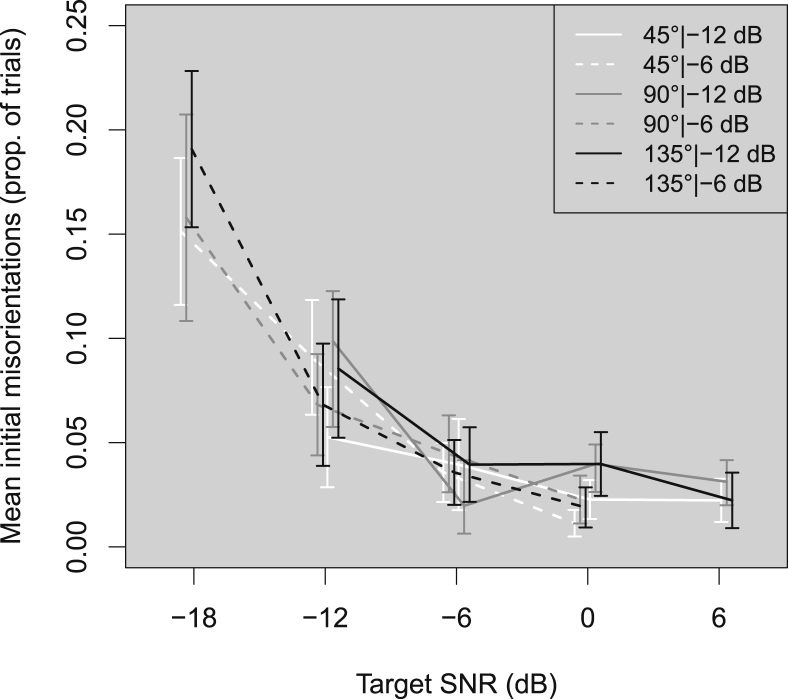
As Fig. 2 but for mean initial misorientations (proportion of trials). Initial misorientations are recorded when the listener initially turns more than 3° in the wrong direction.

Fig. 5 shows initial misorientations (per trial) across listeners as a function of target SNR for each combination of target angle and background level. Overall, initial misorientations increased with decreasing target SNR. The largest increases were between −6 and −18 dB SNR. The increase in fixation error from −12 to −18 dB SNR was significant at 135° target angle, but not at the other angles tested (Bonferroni-corrected *p* = 0.05/3 = 0.0167): 135° (Y_t_ = 0.17 (0.013, 0.33), *p* < 0.0085).

1-way ANOVAs of LME models fitted to the initial misorientation data revealed a main effect of SNR (χ^2^(2) = 29.42, *p* < 0.001). These results suggest that only SNR affected listeners' likelihood of initially misorienting toward a sound. This effect could be seen for SNRs below −6 dB SNR.

### 3.5 Front-back confusions

**Fig. 6:**
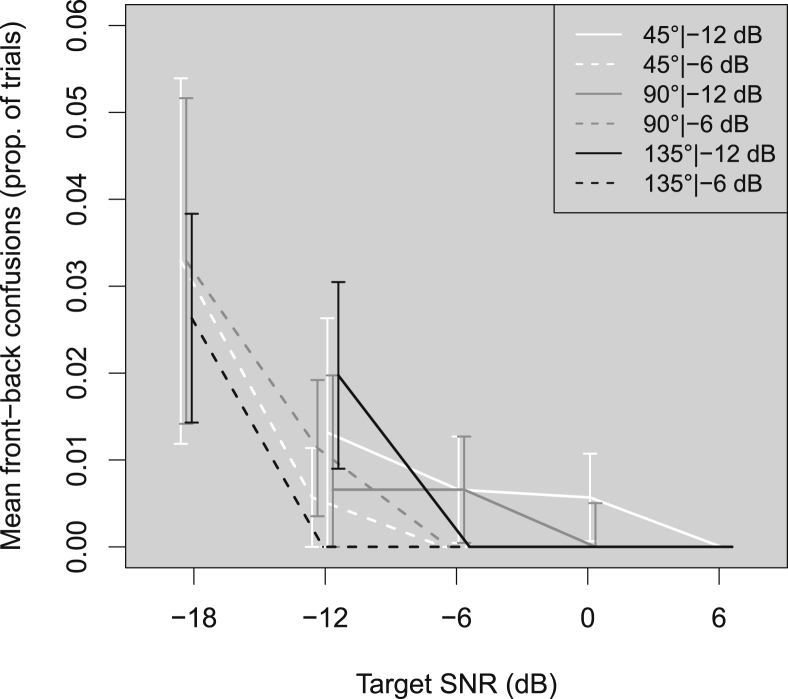
As Fig. 2 but for mean front-back confusions (proportion of trials). Front/back confusions are recorded when the listener ends their orienting movement more than 150° away from the target direction.

Fig. 6 shows the proportion of front-back confusions across listeners as a function of target SNR for each combination of target angle and background level. Overall, front-back confusions were very rare. This was to be expected in an experiment where listeners were allowed to move their heads, as the cues obtained from head movement resolve front-back confusions. Front-back confusions increased at target angle 45° and 90° for −18 dB SNR. At 135°, front-back confusions increased from −6 dB SNR at −12 dB background level, and only increased below −12 dB at the −6 dB background level.

1-way ANOVAs of LME models fitted to the front-back confusion data revealed main effects of target SNR (χ^2^(2) = 10.43, *p* = 0.0054) and background level (χ^2^(1) = 7.94, *p* = 0.0048). These results suggest that at lower levels and target SNRs, targets presented in the rear hemifield may lead to more front-back confusions, but these confusions remain rare even at the lowest SNRs.

The individual results revealed that the front-back confusions were produced by 6 listeners, of whom only one showed front-back confusions on more than 3% of trials. No trends were observed for these listeners with respect to hearing loss, age or duration of hearing-aid use. Front-back confusions occurred throughout the blocks of trials.

### 3.6 Listener hearing loss and age

Exploratory, post-hoc, pairwise correlations were calculated using responses across all target angles in high background noise (−6 dB), −6 dB SNR only. As the data were not normally distributed, Spearman's rank-order correlation coefficient, r_s_, was calculated. Correlations with hearing loss and age were treated as separate analyses. Therefore, the Bonferroni-corrected significance level was set at p = 0.05/6 = 0.008. No significant correlations were found across the metrics for hearing loss or age.

## 4 Discussion

### 4.1 Fixation error

At high target SNRs, fixation error was relatively low across all target angles and background noise levels. Orientation became less accurate as the SNR decreased. The decrease in accuracy with decreasing SNR was steeper for larger angles, which paralleled previous work demonstrating that localization accuracy decreased with decreasing target SNR (Lorenzi et al., 1999, Best et al., 2011). Best et al. (2011) hypothesized that inaudibility of high frequencies and reduced signal detection ability (due to effects of a damaged cochlea) in HI listeners were possible causes.

Duration and initial misorientations increased with decreasing target SNR. The results suggested that uncertainty had a cumulative effect – increasing the angular distance to the target increased the movement duration and the range of angles over which the listener could search, leading to reduced localization accuracy. Another possible and not mutually exclusive explanation is that the reduction in accuracy was due to something akin to listener fatigue – after moving further and for a longer duration than in other conditions, the listeners simply turned ‘close enough’ to the target angle and did not carry out the smaller positional adjustments that they undertook in other, perhaps subjectively ‘easier’, conditions. The evidence for this lay in the reversal results, for which fewer small adjustments to location were made the larger the target angle. However, the reduction in reversals could have been due to listeners knowing that they only had 6 s to orient towards the target, and since larger target angles took longer to turn to, they knew they had less time to make fine adjustments to their position before the next trial started.

The absolute level of the background noise and the target also influenced accuracy. Below 0 dB target SNR, targets presented in −12 dB background noise were less accurately localized than targets at the same SNR presented in −6 dB background noise. This again suggested that audibility, independent of detection in noise, was a factor in successfully orienting towards a sound (Byrne et al., 1992).

### 4.2 Movement duration and reversals

The increase in duration of movement with increasing target angle was perhaps the easiest effect to explain; targets at larger angles required listeners to move further than targets at small angles. Decreasing the target SNR or level also increased movement durations. Accuracy results in these conditions suggested that localization cues became less salient, resulting in less direct and more variable movement towards a target in increasing noise, or when the overall levels of the target and noise were reduced.

Interestingly, movement durations for each target angle appeared to converge at the lowest target SNR (−18 dB), suggesting that once localization cues became sufficiently difficult to use, all positions took equally long to move to. This may have important applications for hearing-aid programs, as it suggests that at low enough SNRs, using acoustic cues alone even targets that are within the listener's field of view take several seconds to orient towards, time which could be vital to the listener to understand and follow a dynamic group conversation.

In a reversal of the trend seen with other metrics, reversals were *more* common at smaller angles. This suggested the use of a finer locating strategy for small angles than for larger angles. These results support the idea that at larger angles, listeners turned to be ‘close enough’ to the target, whereas at small angles, they were more likely to make the small corrections or reversals necessary to orient towards a target more accurately. An alternative explanation is that, on average, targets were further away than 45°, so listeners tended to make a large initial movement, which led to overshooting and then correction in the 45° conditions. Also, listeners had more time to make small adjustments to their position for a target at 45° than for a target at 135° during a 6 s presentation of the target. Reversals were independent of the target SNR or background noise level down to −12 dB. The increase of one and a half to twice the number of reversals at −18 dB SNR suggests that listeners were not orienting per se, but actively *searching* for the target in the noise, turning back and forth until they found it.

### 4.3 Initial misorientations

Decreases in target SNR or increases in background noise increased initial misorientations. Initial misorientations began to increase below −6 dB SNR, similar to the SNR effects observed for accuracy, and duration. Below −6 dB SNR, the noise may have sufficiently reduced the salience of localization cues to require listeners to move their heads and perhaps obtain more information on the position of the source by tracking the relative movement of the target. The lack of variation of initial misorientations with target angle or background noise suggests that the size and audibility of the localization cues (ILDs and ITDs being larger for 90° than 45° or 135°) did not help to reduce them. This differed from results obtained from HI listeners wearing hearing aids (Brimijoin et al., 2014), where initial misorientations increased linearly with increasing target angle, up to 150°.

### 4.4 Front-back confusions

Front-back confusions were very rare, as listeners were allowed to move their heads. The few confusions that did occur were driven by 6 individual listeners, only one of whom showed front-back confusions on more than 3% of trials. We were unable to determine a difference in any of these listeners' attributes that could be responsible for this effect. The front-back confusions were not due to initial acclimatization or learning effects, as the confusions occurred throughout the blocks of trials.

### 4.5 Audibility and age

As described earlier, some data points were removed from the analysis as some of the stimuli were not audible. Without these data points in the analysis, no correlation was found between listeners' orienting behaviour and their hearing loss. This suggests that orienting behaviour may be a part of listening that is independent of hearing loss. However, these findings should be treated with caution, as no attempt was made to control for hearing loss or age during the recruitment of the listeners. In addition, since listeners with greater hearing losses were not included in the results for the lower target SNRs, it is possible that these omissions also reduced the correlation. However, previous analyses that included these listeners also showed no correlation with hearing loss. The differences in behavioural response between NH and HI listeners reported by Brimijoin et al. (2010) were not reproduced. Possible reasons for this include the differences in the task, as ours had no visual component, and the fact that all listeners were hearing impaired.

A limitation of the study was that, unlike hearing aids with directional microphones, no frequency-selective amplification was provided to compensate for the hearing losses of the listeners. This means that the type of hearing loss each listener had may have influenced the results. This effect may have been especially important for bilateral users who were used to receiving most of their acoustic information through a hearing aid with frequency-selective amplification. Further work is required to investigate the effect of amplification on the minimum monitoring SNR.

### 4.6 General discussion

Movement durations and initial misorientations increased as the target SNR decreased below 0 dB, fixation errors increased as the SNR decreased below −6 dB, while reversals and front-back confusions only increased for SNRs below −12 dB. Increasing the target angle increased duration at all SNRs, increased fixation error for SNRs below 0 dB at 135°, decreased reversals above −12 dB SNR, and had no effect on initial misorientations. Decreasing the background noise level (and therefore the absolute level of the target) increased fixation errors and duration below −6 dB SNR. These results suggest that listeners experienced some difficulty orienting towards sources as the SNR dropped below −6 dB.

Across the metrics used, keeping level constant and increasing the background noise level by 6 dB had little or no effect on the results, until the target level was −24 dB relative to the reference source (see the difference between −12 dB target SNR/−12 dB background level and −18 dB target SNR/−6 dB background level). This provided further evidence that restoration of audibility reduces much of the localization deficit observed for HI listeners (Noble et al., 1994).

These results lead to a number of considerations in relation to the design of an optimally directional hearing-aid microphone. The output of a directional microphone could be reduced by as much as 12 dB at 45° relative to 0° with little reduction in a listener's orienting performance across angles, as long as the background noise level was no more than 6 dB above the target level. This raises the possibility of using microphones with higher directionality than are currently used clinically (Brimijoin et al., 2014).

At lower levels and SNRs, orienting performance did not decrease dramatically until the SNR dropped below −12 dB. The fixation error at −12 dB SNR was 5°–15° larger than at positive SNRs, placing the listener within approximately 30° of the target position. In a real-world scenario, this would be sufficient for the listener to see the talker of interest and more importantly to use visual cues, such as lip reading (Grange and Culling, 2016), to help them follow a dynamic group conversation. In addition, the peak output of a directional microphone when in a hearing aid on the head is shifted away from 0°, due to head shadow effects, by 20°–30° towards the side of the ear on which the hearing aid is worn (Brimijoin et al., 2014). This means that listeners should, in theory, look at talkers with their head pointing slightly away from them.

The movement duration increased by approximately 0.5 s for every 6 dB drop in SNR between 0 and −12 dB SNR. Half a second could be of vital importance for listeners to understand what a new talker is saying, given that orienting to a source at 45° can take 3 s at positive SNRs. This constraint could become more critical the more strongly directional the microphone.

The increase in initial misorientations below 0 dB SNR suggested that although the listener may be able to orient towards a source down to −6 or −12 dB SNR, the difficulty and perhaps the effort required to do this increased relative to positive SNRs, which could increase listener fatigue in noisy situations.

The lack of correlation of any of the metrics with hearing loss or age was surprising, and suggested that listener orientation behaviour was not affected by hearing loss or age. A possible caveat to this was that hearing loss and age were confounded in this cohort of listeners, and recruitment that controlled for these factors might reveal an effect. By using a cognitive test, such as the widely used Mini-Mental State Examination (Folstein et al., 1975), other confounding factors may be observed in future studies.

Our use of a sound-treated but not anechoic room minimized the impact of reverberation. Late reverberant reflections continuing beyond the limits of the precedence effect have been found to be detrimental to listeners' localization accuracy (Hartmann, 1983). Therefore, in a reverberant environment, we would expect fixation errors to increase, and the increased uncertainty should manifest itself as increased numbers of initial misorientations and reversals, and increased movement durations.

### 4.7 The minimum monitoring SNR

Some similarities across the metrics were observed that pointed towards a minimum SNR at which orienting behaviour was minimally affected. Performance was worst across all metrics at the lowest SNR (−18 dB). Some metrics, such as reversals, were not significantly different between −12 and 6 dB SNR, which suggests that an SNR of −12 dB was sufficient to stop listeners making small corrections to their head movements. Above −12 dB SNR, the target angle had a large effect on reversals, although in the opposite direction to other metrics, as the 45° target angle produced the largest number of reversals while the fewest occurred at 135°. In comparison, movement duration increased steadily with decreasing target SNR, which suggests that the minimum SNR for which movement duration is unaffected may be above 6 dB. Movement duration was unsurprisingly strongly affected by target angle – the larger the angle, the longer the movement duration. Between these two extremes were fixation errors, and initial misorientations. Apart from the −12 dB background/135° target angle condition, these metrics changed with decreasing SNR between −12 and −6 dB. Therefore, taking all the metrics as a measure of listener orienting behaviour, the minimum monitoring SNR for HI listeners is between −12 and −6 dB, with highly detrimental effects below −12 dB.

### 4.8 Implications for directional hearing-aid microphones

Directional microphones have been one of the more successful strategies employed by hearing aids to increase speech intelligibility, at least in low reverberation situations where the target was presented from directly in front of the listener and spatially separated from the noise sources (Cord et al., 2004). In theory, if the source of interest were directly in front of the listener, then a highly directional microphone pattern would produce the best speech intelligibility. However, in addition to the increased internal noise that such directional microphones produce (Chung, 2004), high directionality may cause problems in dynamic listening environments such as a lively group conversation. A highly directional microphone may reduce the ability to locate a new talker (using auditory cues alone), or to locate them quickly enough to follow a conversation. This may place a higher load on the listener to ‘fill in the gaps’ for the speech they didn't hear, perhaps impairing their abilities more than an omnidirectional microphone in the same setting.

For these reasons, and because of the design limitations placed on microphones due to the size and power consumption requirements of hearing aids (Kates, 2008), the directionality of hearing-aid microphones has been limited. Occasionally there can be less than a 2 dB difference in directivity index between omnidirectional and directional settings on the same hearing aid (Brimijoin et al., 2014).

Grange and Culling (2016) have assessed the head-orientation benefit for speech intelligibility in noise. A model of spatial release from masking predicted, and subsequent psychoacoustic tests confirmed, that orienting one's head 30° away from a talker improved speech intelligibility by 2–5 dB for NH listeners and bilateral and unilateral cochlear-implant users. Importantly, a head orientation of this magnitude would not affect lip-reading benefit, as the eyes can move up to 45° (Guitton and Volle, 1987), though it may feel unnatural to turn one's head to this degree during a conversation. A 2-dB head orientation benefit was measured for NH listeners in a realistic restaurant scenario, comparable to the best real-world benefit provided to HI listeners by adaptive directional microphones (Woods and Trine, 2004). The benefits and deficits produced by dynamically changing head orientation have yet to be investigated.

Hearing-aid design should be compatible with listener behaviour, and people generally move their heads in typical social situations to look at the person talking (Kendon, 1967), although this is not possible for example when sitting next to someone eating a meal, or looking at a conference poster while a presenter explains it. Directional-microphone design for hearing aids, therefore, must be a compromise between the need to reduce noise when attending to a single source, the audibility and localizability of off-axis sources in multi-talker environments, and the physical constraints put on hearing aids by their limited size, processing power and power consumption (Kates, 2008). Technology now exists to alter the directionality of hearing-aid microphones based not only on the acoustic signals picked up by the microphones, but on head and eye movement (Zohourian et al., 2015, Kidd et al., 2013). These systems have used head and eye movements to alter the response patterns of adaptive directional microphones during simple head movements. Other systems have improved direction of arrival estimation for sound sources by compensating for (Boyd et al., 2013) and utilizing (Archer-Boyd et al., 2015) head movement. It has been shown that systems that select talkers based on eye position can improve recall after listening to a passage of speech, in comparison to omni-directional hearing aids (Hart et al., 2009).

Best et al. (2016) have developed a speech test for dynamic, multi-talker situations that better reflects real-world listening scenarios. What is now required are detailed analyses of head and eye movement during dynamic conversations. These analyses will provide information on several unknown quantities for head and eye-controlled adaptive directional microphones, such as: what are the types and ranges of head/eye movements made during conversations; how much individual/cultural variation is there; and how much does the conversation setting (social and acoustic) and configuration of the listeners affect head movement? An immediate issue raised by this study is the range of angles over which the maximum recommended attenuation of 12 dB should be applied. This may depend on the position of talkers around the listener.

## 5 Conclusions

The findings presented in this study should serve as initial guidelines for future work, both in conversation analysis and dynamic spatial signal processing for hearing aids. Our results suggest that: 1) if one intends to make a directional microphone that is usable in a dynamic conversation, then off-axis attenuation should be no more than 12 dB, and 2) a directional hearing-aid microphone that is adapted based on information about head movement might be able to provide benefit, with a narrow, ‘torch in the dark’, microphone response being used when the listener is attending to a source directly in front of them, opening up to a wider, less directional response when the listener moves their head to find the next target of interest.
